# Different mechanisms underlie compulsive alcohol self-administration in male and female rats

**DOI:** 10.1186/s13293-024-00592-5

**Published:** 2024-02-17

**Authors:** Sanne Toivainen, Li Xu, Francesco Gobbo, Andrea Della Valle, Andrea Coppola, Markus Heilig, Esi Domi

**Affiliations:** 1 Center for Social and Affective Neuroscience, Department of Biomedical and Clinical Sciences, Linköping University, S-581 85, Linköping, Sweden; 2 Centre for Discovery Brain Sciences, University of Edinburgh, 1 George Square, Edinburgh, EH8 9JZ UK; 3 Present Address: School of Pharmacy, Center for Neuroscience, University of Camerino, 62032 Camerino, Italy

**Keywords:** Sex differences, Alcohol, Compulsivity, Operant self-administration, Motivation, Stress

## Abstract

**Background:**

Sex is an important factor in the progression and treatment of alcohol addiction, and therapeutic approaches may have to be tailored to potential sex differences. This highlights the importance of understanding sex differences in behaviors that reflect key elements of clinical alcohol addiction, such as continued use despite negative consequences (“compulsive use”). Studies in experimental animals can help provide an understanding of the role sex plays to influence these behaviors.

**Methods:**

Large populations of genetically heterogeneous male and female Wistar rats were tested in an established model of compulsive alcohol self-administration, operationalized as alcohol responding despite contingent foot shock punishment. We also tested baseline (fixed ratio, unpunished) operant alcohol self-administration, motivation to self-administer alcohol (progressive ratio), and temporal discounting for alcohol reward. In search of predictors of compulsivity, animals were screened for novelty-induced place preference, anxiety-like behavior, pain sensitivity and corticosterone levels. The estrous cycle was monitored throughout the study.

**Results:**

Unpunished self-administration of alcohol did not differ between males and females when alcohol intake was corrected for body weight. Overall, females showed higher levels of compulsive responding for alcohol. Compulsive response rates showed bimodal distributions in male but not in female rats when intermediate shock intensities were used (0.2 and 0.25 mA); at higher shock intensities, responding was uniformly suppressed in both males and females. We also found less steep discounting in females when alcohol was devalued by delaying its delivery. Males exhibited a stronger motivation to obtain alcohol under unpunished conditions, while females showed higher corticosterone levels at baseline. Factor analysis showed that an underlying dimension related to stress and pain predicted compulsivity in females, while compulsivity in males was predicted by a reward factor. We did not find differences in alcohol-related behaviors throughout the various stages of the estrous cycle.

**Conclusions:**

Our results suggest that mechanisms promoting compulsivity, a key feature of alcohol addiction, likely differ between males and females. This underscores the importance of considering sex as a biological variable in both preclinical and clinical research, and has potential treatment implications in alcohol addiction.

**Supplementary Information:**

The online version contains supplementary material available at 10.1186/s13293-024-00592-5.

## Plain language summary

Sex plays an important role in the progression and treatment of alcohol addiction. While men show a higher prevalence of alcohol addiction, women are more susceptible to the adverse effects of excessive alcohol consumption. Additionally, women often rely on heavy drinking as a maladaptive coping mechanism to alleviate stress and anxiety, driven by negative affect. On the other hand, men are more likely to report heavy drinking and relapse in response to positive emotions and social influences. These sex-based differences underline the importance of understanding how vulnerability to alcohol addiction and its treatment varies in males and females.

We used genetically heterogeneous rats to explore the behavioral traits that contribute to compulsivity, a key clinical feature of alcohol addiction. We found that motivation to self-administer alcohol was higher in males, while females showed higher compulsive alcohol self-administration. In males, motivation to self-administer alcohol showed a significant correlation with compulsivity, while in females compulsivity was predicted by higher basal corticosterone levels.

These findings underlie the importance of sex-specific factors in compulsive alcohol self-administration, with potential prevention and treatment implications in alcohol addiction.

**Supplementary Information:**

The online version contains supplementary material available at 10.1186/s13293-024-00592-5.

## Highlights

- Male rats showed a higher motivation to obtain alcohol.
- Females showed higher levels of compulsive responding for alcohol and a less steep discounting when alcohol was devalued by delaying its delivery.
- In males compulsivity was predicted by a reward factor, while in females by stress-pain factors.

**Supplementary Information:**

The online version contains supplementary material available at 10.1186/s13293-024-00592-5.

## Introduction

Sex plays a critical role in the etiology and treatment of drug and alcohol addiction. Despite a higher prevalence of alcohol addiction in men [1], women are more vulnerable to the negative effects of excessive alcohol use, including alcoholic liver problems and alcohol-related cancers [2]. In addition, alcohol-dependent women report higher rates of comorbid psychiatric conditions such as anxiety and mood disorders [3]. As a major factor promoting relapse in women is relying on heavy drinking as a maladaptive strategy to alleviate negative affective states [4–7]. In contrast, men often report heavy drinking and relapse in response to positive emotions and social factors [8–10]. Taken together, this suggests potential sex differences in the mechanisms behind progression into, and maintenance of alcohol addiction. If present, these differences may in turn have important therapeutic implications.

Until recently, preclinical studies on alcohol addiction have mostly been conducted on males, and only limited data are available to allow an understanding of potential sex differences. In rodent models, it has generally been found that females consume more alcohol than males [11–16], but results have differed depending on strain and behavioral paradigms. Under low-effort voluntary drinking conditions (i.e., home cage drinking with intermittent or continuous access), females have been reported to consume more alcohol than males [13, 15]. However, in operant models, several studies have demonstrated mixed results showing no difference or increased alcohol intake in females depending also on the genetic background [17–19]. Using a conditioned place preference paradigm, female rats have been shown to be more sensitive to the rewarding effect of alcohol when compared to ovariectomized females or male rats, implying that ovarian hormones and the estrus cycle can affect the rewarding effects of alcohol [20].

Rodent models of aversion-resistant drug taking attempt to capture a dimension of addictive disorders that is in part separate from simple drug use. These models capture a defining feature of clinical addiction, i.e., continued use despite negative consequences, commonly referred to as “compulsivity” [21]. Studies in this type of models report that females consume more alcohol when alcohol delivery is paired with footshock [22], or when alcohol is adulterated with quinine [23–26]. Nevertheless, in alcohol-preferring (P) rats, data showed that prolonged alcohol intake led to increased resistance to quinine adulteration specifically in males [27]. While, in a different study, it has been reported that females display more aversion-resistant alcohol seeking than males in a modified place aversion test [28].

We recently used alcohol self-administration that is resistant to footshock punishment to study individual differences in punishment-resistant alcohol self-administration [29, 30]. We found that, among outbred male Wistar rats, a subpopulation continued to self-administer alcohol despite a contingent electric footshock, and could thus be operationally classified as “compulsive”. The emergence of compulsivity was not the result of differences in alcohol exposure or sensitivity to shock, and generalized to another model of aversion-resistance, quinine adulteration. In line with this finding, punishment of alcohol reinforced responding in alcohol-preferring P-rats shows a bimodal distribution of the population [31]. It is currently unknown whether females show similar individual variation in punishment-resistant alcohol self-administration.

Here, we therefore used Wistar rats to study the potential role of sex as a biological variable in a battery of alcohol-related behaviors comprising both unpunished and punished (compulsive) operant alcohol self-administration, motivation to self-administer alcohol, and delay discounting of alcohol reward. Individual variation in punishment-resistant alcohol self-administration in males and females was assessed across increasing punishment intensities. In search of traits predictive of compulsivity, animals were screened for novelty-induced place preference, anxiety-like behavior, pain sensitivity and corticosterone levels. The estrous cycle was monitored throughout the study and additional control experiments were conducted to elucidate the role of sex in alcohol-related behaviors.

## Materials and methods

### Animals

Adult (7–9 weeks) male (*n* = 32) and female (*n* = 32) Wistar rats (Charles River, Germany) weighing ~350 g and 230 g, respectively, at the beginning of the experiments were group housed with ad libitum access to tap water and food pellets. Rats were single housed during the two-bottle choice drinking paradigm. Animals were maintained in a temperature- and humidity-controlled vivarium on a 12-h light/dark cycle (lights off at 7:00 a.m.). Body weights were monitored weekly, and rats were handled three times before the start of the experimental procedure. All experiments were performed during the dark phase of the light–dark cycle. Experimental procedures were conducted in accordance with the European Union Directive 2010/63/EU, and the protocol was approved by the Ethics Committee for Animal Care and Use at Linköping University.

### Novelty-induced place preference

Novelty-induced place preference was performed using two identical arenas formed by two compartments (30 × 30 × 45 cm) connected by a central rectangular corridor (30 × 10 cm large, 45 cm high) with two opposite openings (8 cm × 4 cm). Openings to the central corridor could be blocked by sliding panels [32]. To add visual and sensory differences between the compartments, each compartment had a wall made of transparent Plexiglass, and the floor grids were of different shapes (circles or squares). The lighting was a low-intensity red light, and the arenas were placed to ensure even illumination. Each rat was first given 5 min of exploration in the central corridor and then 20 min of habituation in the assigned familiar compartment. After the habituation period, the rat was placed in the central corridor, after which the dividers to both side-compartments were immediately removed, and the animal was allowed to freely explore the entire arena for 15 min. The behavior was recorded by a digital camera for later manual scoring of: (a) latency time to enter into the novel compartment; (b) time spent in each compartment; and (c) number of entries in each compartment The novelty preference index was defined as the ratio of time spent in the novel compartment versus the total time spent between the novel and the familiar compartment [32].

### Anxiety-like behavior

Anxiety-like behavior was assessed before the punished alcohol self-administration procedure using the elevated plus maze (EPM) as previously described [33]. Briefly, the maze consisted of two open arms (50 × 10 cm) and two closed arms (50 × 10 cm) connected by a central square (10 × 10 cm), with 30-cm-high walls, and elevated 50 cm. The experiment was performed under a dim source of light and the behavior was recorded by a video tracking system and scored manually by two blinded operators. The 5-min test procedure began when the animal was placed in the center of the maze, facing a closed arm. The percentage of time spent exploring the open arms (% Open Time) was used as a measure of anxiety-like behavior, whereas the number of entries into closed arms was used as an indicator of general motor activity.

### Alcohol self-administration

Operant and drug-naïve rats were trained to self-administer 20% (v/v) alcohol without using water deprivation or saccharin/sucrose fading procedures as described previously [34]. Operant training and testing were performed in 48 identical operant chambers (30.5 cm × 29.2 cm × 24.1 cm; Med Associates Inc., St. Albans, VT, USA) housed in sound-attenuating cubicles. Each operant chamber was equipped with two retractable levers positioned laterally to a liquid cup receptacle. Females and males were placed in separate boxes to minimize the influence of olfactory stimuli. Briefly, rats were initially trained on an FR1 5-s time-out (TO) schedule to self-administer 20% alcohol during 30-min sessions, 5 days per week. At the onset of the self-administration session, levers were extended marking alcohol availability.

Each active lever response was reinforced by the delivery of 100 ml of 20% alcohol in water into the drinking receptacle, and activated the concomitant 5-s TO period signaled by illumination of the cue light. During 5-s TO no rewards were given, but responses were monitored. Each inactive lever response was recorded but had no programmed consequences. FR1 training was continued until self-administration levels stabilized with less than 15% changes in total number of reinforcers earned over the last three sessions. Once a stable baseline was reached, the reinforcement ratio was increased to FR2 and the training continued until self-administration rates stabilized again.

### Footshock-punished alcohol self-administration

Compulsivity was operationalized as responding for alcohol when its delivery was associated with a footshock punishment as previously described [29, 35]. Briefly, conditions were identical to baseline self-administration (i.e., 30-min sessions), but each completed FR2 ratio (i.e., 2 responses) was paired with a 0.5-s footshock, contingent with the delivery of 100 µl 20% alcohol in water in the adjacent drinking well. Shock intensities ranging from 0.1 to 0.35 mA over 0.5 s were progressively tested across 10 days for each shock intensity. Compulsivity was indexed by a Resistance Score (RS) for each rat, calculated as: (punished alcohol deliveries)/(punished alcohol deliveries + mean alcohol deliveries of last 3 non-punished sessions) [29, 31]. We calculated a persistence score representing the day-to-day variability of the animals’ daily RS after establishing the rats’ response to punishment. Persistence score was defined taking the average RS for each rat on days 8–10 of 0.20 mA punishment (RS_0.20 mA_) as ${{\text{PS}}}_{0.20 {\text{mA}}}= \sqrt{\frac{{\sum }_{i=0}^{n}{\left({{\text{RS}}}_{i}-{{\text{RS}}}_{0.20 {\text{mA}}}- {\sum }_{i=0}^{n}\left({{\text{RS}}}_{i}-{{\text{RS}}}_{0.20 {\text{mA}}}\right)/n\right)}^{2}}{n}}$, where *n* is the number of days after the 0.20 mA punishment. Analogously, we defined Persistence score (0.25) considering days 8–10 of 0.25 mA punishment as a reference. 0.20 and 0.25 mA intensities were chosen based on the statistical results showing sex differences in response to punishment.

### Quinine adulteration

Male and female Wistar rats were also assessed for aversion-resistant alcohol intake using quinine adulteration. After stable alcohol self-administration under FR2, increasing concentrations of quinine (10, 50, 100, 150, 200 and 250 mg/l) were added to the ethanol (20%). Quinine concentration was increased every 3 consecutive sessions. Resistance to quinine adulteration was assessed as the resistance score in alcohol self-administration after addition of quinine: (quinine-adulterated alcohol deliveries)/ (quinine-adulterated alcohol deliveries + mean alcohol deliveries of the last three baseline sessions).

### Pain sensitivity

Animals were placed in the operant chamber, and footshock was delivered starting at 0.05 mA and increasing shock intensity by 0.05 mA every 30 s. Footshock threshold was defined as a jump with all four paws off the grid as previously described [29].

### Progressive ratio schedule of reinforcement

Motivation to obtain alcohol was measured using a progressive ratio (PR) schedule [36]. PR conditions were identical to baseline self-administration, but with an increased response requirement per alcohol reinforcer according to the following scale: 1, 2, 3, 4, 6, 8, 10, 12, 16, 20, 24, 28, 32… The self-administration session terminated once 30 min had elapsed without a reinforcer being obtained. The breakpoint was defined as the last completed response requirement during the progressive ratio test.

### Temporal discounting of alcohol reward

After footshock punishment, animals were allowed to recover lever pressing for alcohol in the absence of punishment. Once rates had recovered and baseline was re-established, delays (5, 10, 20 and 30 s) were introduced between the second lever response in the presence of the discriminative cue (FR2), and the reward availability. Delayed rewards were fitted to a hyperbolic discounting function (*V* = [1/(1 + k*D)], where *V* is the baseline value of the alcohol reinforcers in the absence of the delay, *D* is the delay in time and *k* is the discounting coefficient. Higher values of k indicate a steeper discounting of reward value as function of time, and are found in people with addictive disorders [37].

### Quinine preference

As a control for taste reactivity, quinine preference was assessed using a two-bottle choice paradigm, where increasing concentrations of quinine were added to one water bottle (0, 10, 25 and 50 mg/l) access [38]. Quinine concentration was increased every 4 days. Bottle positions were alternated to control for potential side preferences and the preference score for quinine was calculated as follows: (volume of adulterated solution consumed)/(volume of adulterated solution + water consumed).

### The estrous cycle

To determine whether hormonal fluctuations across the estrous cycle affected alcohol-related behaviors, vaginal cellular contents were collected on 5 consecutive days throughout the experimental procedures. To avoid experimental stress, sample collection was performed at a minimum of 60 min after the completion of the experiment. Briefly, a sterile cotton swab dampened with sterile saline was inserted into the vagina ~ 4 mm and rotated. The swab was smeared on a microscopy slide and allowed to air dry for the cytological evaluation. Cells were fixed through submersion in 99.5% EtOH for 10 min and then stained in 0.1% Cresyl Violet. Residual stain was washed out using Millipore Milli-Q water. Vaginal cytological analysis was examined under light microscopy to determine the cell types and characterize the cycle phases. These were defined as follows. Diestrus: abundance of leukocytes and presence of few epithelial cells that begin to be detected just prior to transition to proestrus. Proestrus: clusters of round, well-formed nucleated epithelial cells. Estrus: irregular cornified squamous epithelial cells. Metestrus: the presence of small leukocytes, epithelial and cornified cells [39]. For analysis, samples were pooled as either estrus or non-estrus as previously described [40].

### Corticosterone assay

Blood samples were collected from the tail vein following basal and punished self-administration. Samples were centrifuged for 15 min at 4 °C, 2000*g* per minute, and plasma was extracted. Corticosterone was extracted by adding five parts of ethyl acetate (Thermo Fisher Scientific Inc., Waltham, MA, USA) to each plasma sample. The organic solvent layer was then transferred to tubes prefilled with water and then to a second tube following a second separation of phases. This procedure was repeated twice, after which the samples were dried in a vacuum concentrator and samples were redissolved in DetectX. A corticosterone enzyme immunoassay kit (Arbor Assays, Nordic Biosite AB) was used to analyze the samples for corticosterone. Detection levels for corticosterone were 7.8–1000 ng/ml.

### Statistical analysis

**Fig. 1:**
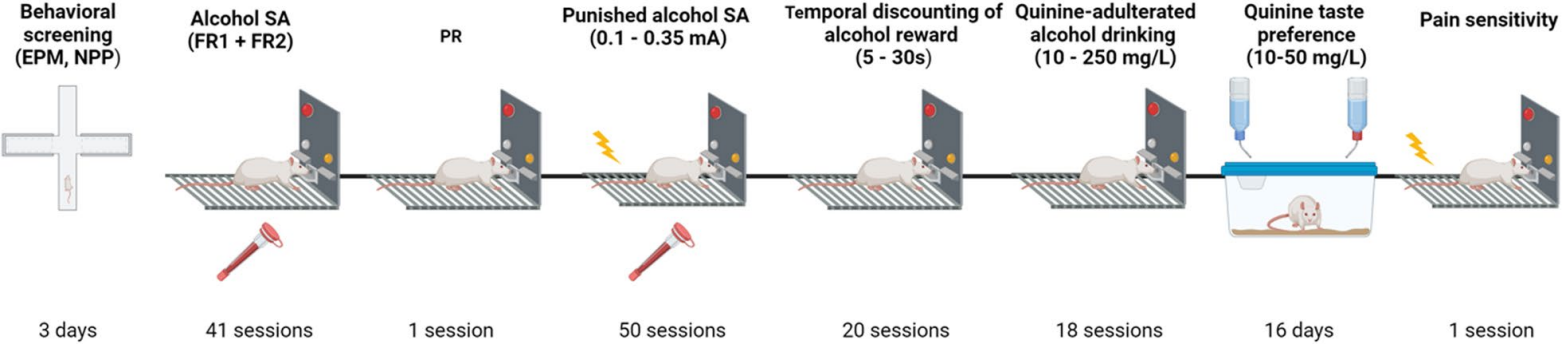
Experimental timeline

Data were analyzed with STATISTICA, Stat Soft 13.0 (RRID:SCR_014213), using analysis of variance (ANOVA), with factors and degrees of freedom for the respective analysis indicated in conjunction with its results. Prior to ANOVA, data were examined for significant violations for assumptions of homogeneity of variance and normality of distribution using Levene’s and Shapiro–Wilk test, respectively. Where homogeneity of variance or normality were significantly violated, data were square root transformed. Statistically significant difference was set at *P* < 0.05. Post hoc analyses were conducted when appropriate using Newman–Keuls test. The data are presented as the mean ± SEM. Hartigans’ dip test for unimodality [41] implemented in UniDip Python package was used to test unimodality of one-dimensional distributions. Experimental timeline is shown in Fig. 1.

## Results

### Male and female rats do not differ in alcohol self-administration when corrected for body weight

**Fig. 2:**
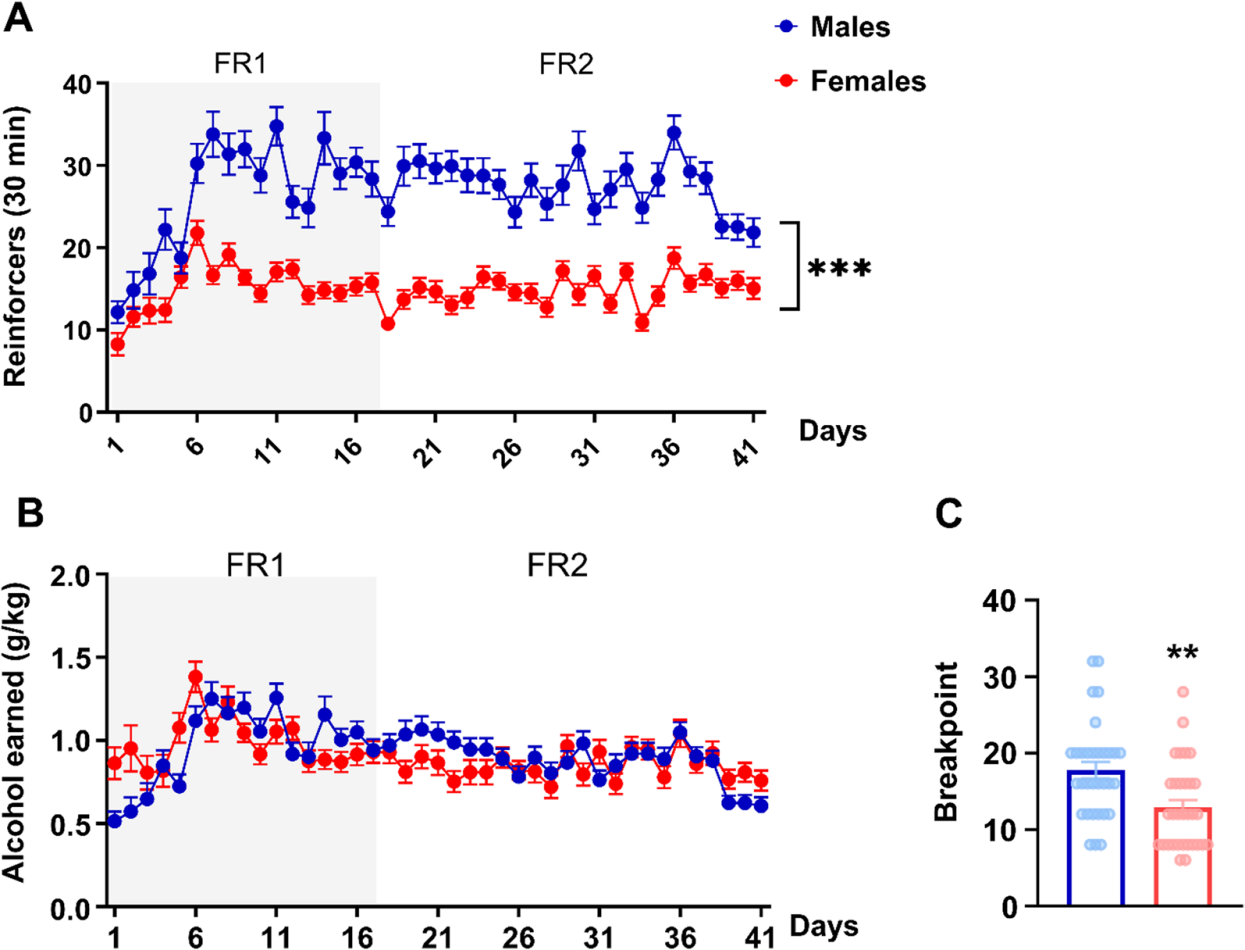
Male and female rats do not differ in alcohol self-administration when corrected for body weight, but males show a higher motivation to self-administer alcohol. **A** Mean reinforcers (± SEM) and **B** mean of alcohol (g/kg) (± SEM) earned during a 30-min alcohol 20% FR2 self-administration). **C** Mean break points (± SEM) reached during a progressive ratio session of 11% alcohol in male and female rats. **p < 0.01, ***p < 0.001

Under unpunished fixed ratio conditions, male rats obtained a significantly higher absolute number of alcohol reinforcers than females (Fig. 2A; repeated measures ANOVA, main effect of session: *F*_40,2480_ = 11, *p* < 0.001; *η*^2^ = 0.12; main effect of sex: *F*_1,62_ = 83.38, *p* < 0.001; *η*^2^ = 0.57; sex × session interaction: *F*_40,2480_ = 4.2, *p* < 0.001; *η*^2^ = 0.06). Newman’s Keuls post hoc analysis showed that males achieved a significantly higher number of alcohol reinforcers than females after one week from the acquisition of alcohol self-administration (*p* < 0.001).

However, when alcohol intake was corrected for body weight, males and females earned the same amount of alcohol (Fig. 2B, repeated measures ANOVA, main effect of session: *F*_40,2480_ = 8.9, *p* < 0.001; *η*^2^ = 0.12; main effect of sex: *F*_1,62_ = 0.032, *p* = 0.859). Although there was a significant sex × session interaction (*F*_40,2480_ = 3.4, *p* < 0.001; *η*^2^ = 0.05), Newman’s Keuls post hoc analysis did not show sex differences in alcohol intake (g/kg) at any individual time point across the sessions. Fixed-ratio, unpunished self-administration did not vary as a function of estrous phase in alcohol self-administration (*F*_1,158_ = 0.32, *p* = 0.57).

### Male rats show a higher motivation to self-administer alcohol

Under the progressive ratio schedule, males showed a higher motivation to self-administer alcohol as demonstrated by elevated break points compared to females (Fig. 2C; *F*_1,62_ = 10.76, *p* < 0.01; *η*^2^ = 0.14). Similar to fixed-ratio self-administration, progressive ratio breakpoints were unaffected by estrous phase (*F*_1,30_ = 1.7, *p* = 0.20).

### Compulsivity is higher in females

Male and female rats (*N* = 32 per sex) were screened for punishment-resistant alcohol self-administration and their resistance to quinine adulteration as previously described [29, 30]. When alcohol delivery was paired with increasing shock intensities (0.1–0.35, 0.5-s shock) or adulterated with increasing quinine concentrations (10–250 mg/l) alcohol self-administration was decreased overall, but with considerable sex differences.

**Fig. 3:**
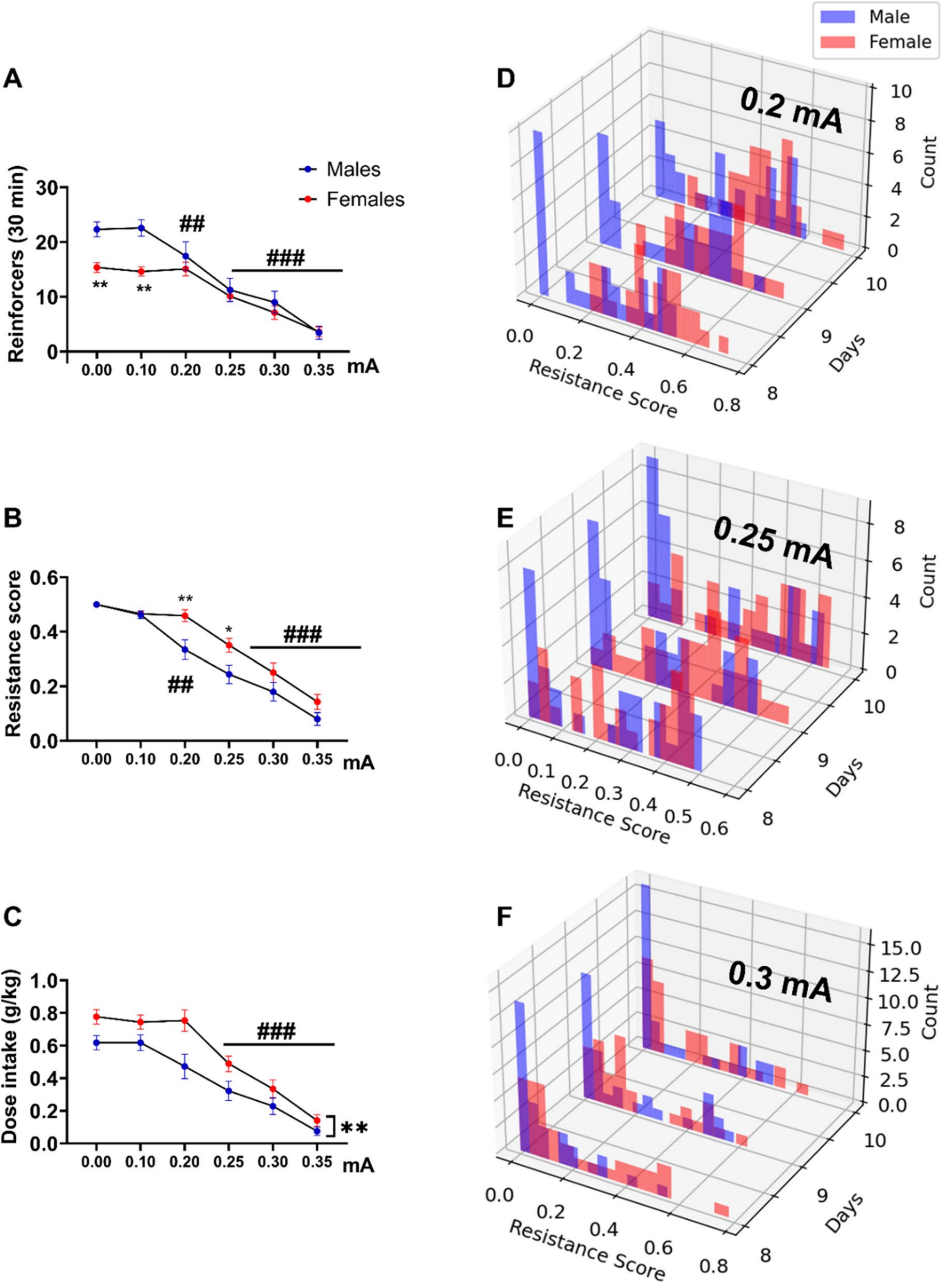
Female rats show a higher resistance to shock-punished alcohol self-administration at progressively increasing shock intensities. **A** Mean reinforcers, **B** resistance score, **C** dose intake (± SEM) earned during 30-min punished self-administration sessions of 20% EtOH (FR2). **D–F** Resistance score distribution of punished alcohol self-administration across 10 days color coded for males (blue) and females (red) under increasing shock intensities (0.2, 0.25 and 0.3 mA). ^##^*p* < 0.01, ^###^*p* < 0.001, **p < 0.01

When alcohol delivery was paired with a 0.1-mA shock, male rats achieved a higher number of alcohol reinforcers compared to females (Fig. 3A, repeated measures of ANOVA main effect of sex: *F*_1, 62_ = 4,38, *p* = 0.04; *η*^2^ = 0.06; shock: *F*_5, 310_ = 66.6; *p* < 0.001; *η*^2^ = 0.52; sex x shock interaction *F*_5, 310_ = 4.6; *p* < 0.001; *η*^2^ = 0.07). Newman’s Keuls post hoc analysis showed that males achieved a significantly higher number of alcohol reinforcers under unpunished conditions and when alcohol delivery was associated with 0.1 mA shock compared to females (*p* < 0.01). Male and female rats significantly decreased the number of punished alcohol reinforcers compared to their baseline at 0.2 and 0.25 mA shock, respectively. Under conditions of increasing punishment intensity, female rats showed a higher resistance score and consumed more alcohol compared to males (Fig. 3B, C, repeated measures of ANOVA on the resistance score; main effect of sex: *F*_1,62_ = 6.2, *p* = 0.01, *η*^2^ = 0.09; shock: *F*_5,310_ = 97, *p* < 0.001; *η*^2^ = 0.61; interaction sex × shock: *F*_5, 310_ = 2.66, *p* = 0.03; *η*^2^ = 0.04; alcohol intake (g/kg): main effect of sex: *F*_1, 62_ = 8.14, *p* = 0.005; *η*^2^ = 0.11; shock: *F*_5,310_ = 78.28, *p* < 0.001; *η*^2^ = 0.55; interaction: sex × shock: *F*_5,310_ = 1.86, *p* = 0.1). Post hoc tests showed a significant decrease of the resistance score in males and females starting at 0.2 (*p* < 0.01) and 0.25 mA (*p* < 0.001), respectively, with female rats showing significantly higher resistance scores than males at 0.2 and 0.25 mA shock (*p* < 0.01 and *p* < 0.05, respectively).

We next examined the population distribution of punishment resistance scores in males and females, obtained over 10 self-administration sessions under each shock intensity. As previously reported in Wistar males [29], under basal conditions, male and female rats were unimodally distributed, *D* = 0.062, *p* = 0.47 and *D* = 0.0625, *p* = 0.41, respectively. When alcohol delivery was paired with a 0.1-mA, 0.5-s shock, the population was unimodal in both male (*D* = 0.062, *p* = 0.42) and female rats *D* = 0.06, *p* = 0.47). In a replication of our prior findings, the population distribution became bimodal in male rats under 0.2 and 0.25 mA punished alcohol self-administration sessions from session 6 onward (Fig. 3D, E; Hartigans’ dip test *D* = 0.1, *p* = 0.002; *D* = 0.09, *p* = 0.009). In contrast, females continued to be distributed unimodally.

Under conditions of increasing punishment intensity (0.3, 0.35), male rats again became unimodally distributed (Fig. 3F; Additional file 1: Fig. S1). Females remained unimodally distributed under all punishment conditions. Male rats showed a significantly more stable resistance at 0.2 and 0.25 mA as shown by a lower persistence score value (St Dev), indicating higher consistency in their response across days (see “Methods”) (Additional file 1: Fig. S2).

Shock sensitivity differed between male (*N* = 16) and female (*N* = 16) rats, with females being significantly more sensitive to the shock (Additional file 1: Fig. S3A, *F*_1,30_ = 23.9, *p* < 0.001; *η*^2^ = 0.44). Thus, the higher punishment-resistant alcohol self-administration in females cannot be explained by a decreased shock sensitivity.

### Female rats also show a higher resistance to quinine adulteration

**Fig. 4:**
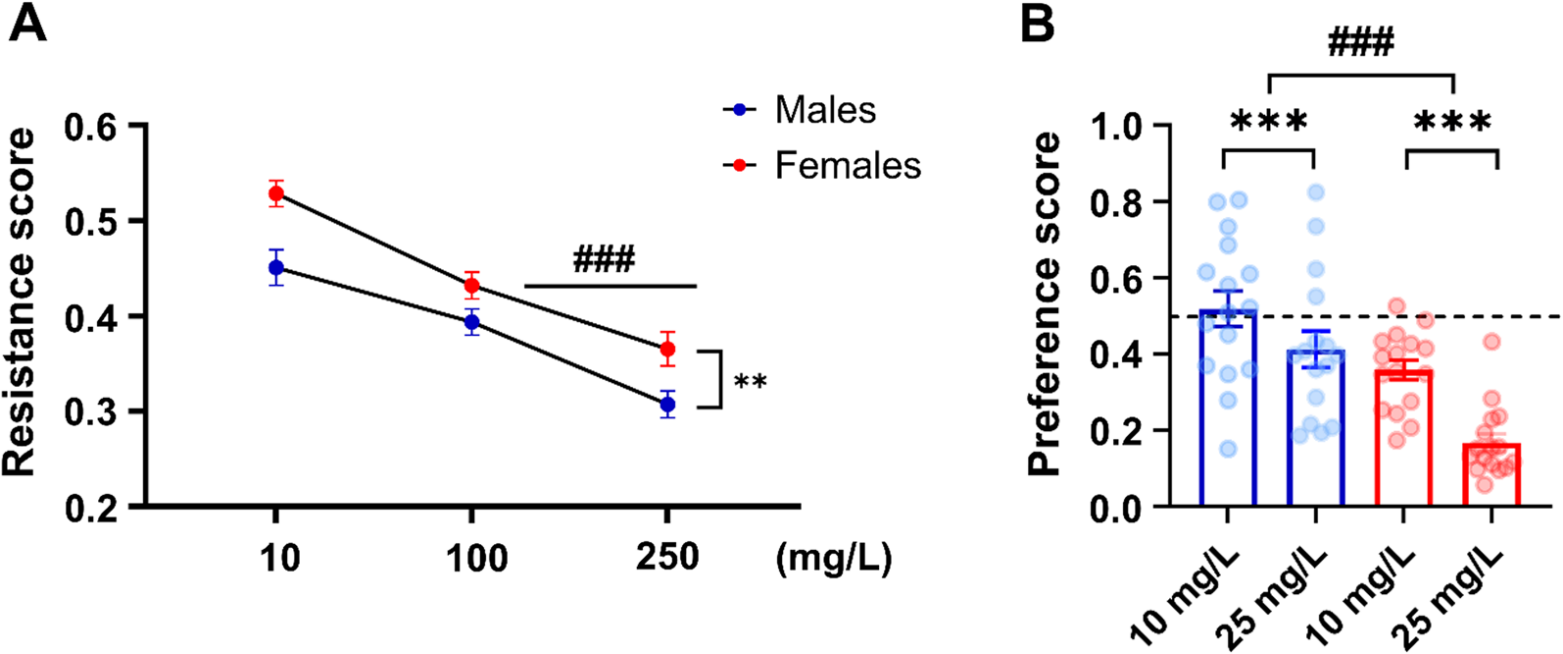
Female rats show a higher resistance to quinine-induced alcohol adulteration.** A** Resistance score (± SEM) obtained during 30-min quinine-adulterated alcohol self-administration (10, 100, 250 mg/l). **B** Preference score for quinine-adulterated water. ^###^*p* < 0.001, **p < 0.01, ***p < 0.001

Resistance to consuming alcohol despite adverse consequences was additionally assessed using quinine adulteration [42, 43]. Male and female rats were allowed to recover their alcohol lever pressing after removing the footshock. When alcohol was adulterated with quinine (10, 100 and 250 mg/l), resistance scores decreased significantly in both sexes at increasing quinine concentrations, and consistent with the shock-punishment results, females demonstrated a significantly higher resistance to consuming alcohol despite quinine adulteration compared to males (Fig. 4A, repeated measures of ANOVA, main effect of sex: *F*_1,62_ = 10.1, *p* = 0.02; *η*^2^ = 0.14; dose: *F*_2,124_ = 105,64, *p* < 0.001; *η*^2^ = 0.63; interaction dose × sex *F*_2,124_ = 1,79, *p* = 0.2). As a control for taste reactivity to quinine, consumption of and preference for water and quinine were measured using a two-bottle free-choice continuous access in male (*N* = 16) and female rats (*N* = 16) [38]. Female rats showed a higher sensitivity to quinine-adulterated water compared to male rats (Fig. 4B, repeated measures ANOVA, main effect of sex: *F*_1,30_ = 24.2, *p* < 0.001; *η*^2^ = 0.44; main effect of concentration: *F*_1,30_ = 41.5, *p* < 0.001; *η*^2^ = 0.58 with no significant interaction sex × concentration: *F*_1,30_ = 1.7, *p* = 0.2). Post hoc analysis showed that females had a significantly lower quinine preference compared to males at both doses, *p* < 0.01 and *p* < 0.00, respectively. Thus, the higher resistance to reducing consumption of alcohol when the solution was adulterated with quinine observed in females cannot be explained by differences in sensitivity to the bitter taste of quinine.

### Females show lower temporal discounting rates of alcohol reinforcers

**Fig. 5:**
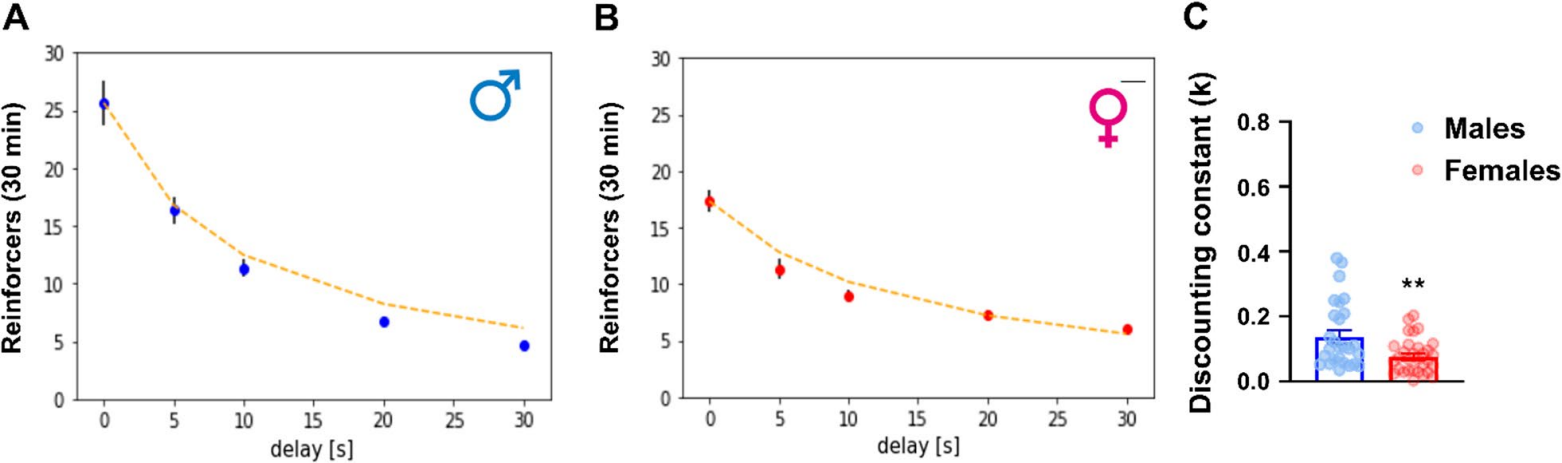
Female rats show less steep temporal discounting of alcohol rewards. **A, B** Fitted hyperbolic of mean reinforcers (± SEM) in 30-min delayed alcohol self-administration and **C** discounting constant for males and females **p < 0.01

We next examined the sensitivity to delaying the alcohol reward in male (*n* = 32) and female (*n* = 32) rats. Delays (5, 10, 20 and 30 s) were introduced between the second lever response in the presence of the discriminative cue (FR2), and the reward availability. Males had significantly higher *k* discounting values, reflecting a steeper discounting of delayed alcohol rewards. Four males were excluded due to being significant outliers (Fig. 5A–C, one-way ANOVA, main effect of sex, *F*_(1,58)_ = 8.1; *p* < 0.01; *η*^2^ = 0.12). We did not find differences in sensitivity to delay between compulsive (RS > 0.45) and non-compulsive (RS < 0.45) animals within sex (data not shown).

### Predictors of compulsivity in male and female rats

**Fig. 6:**
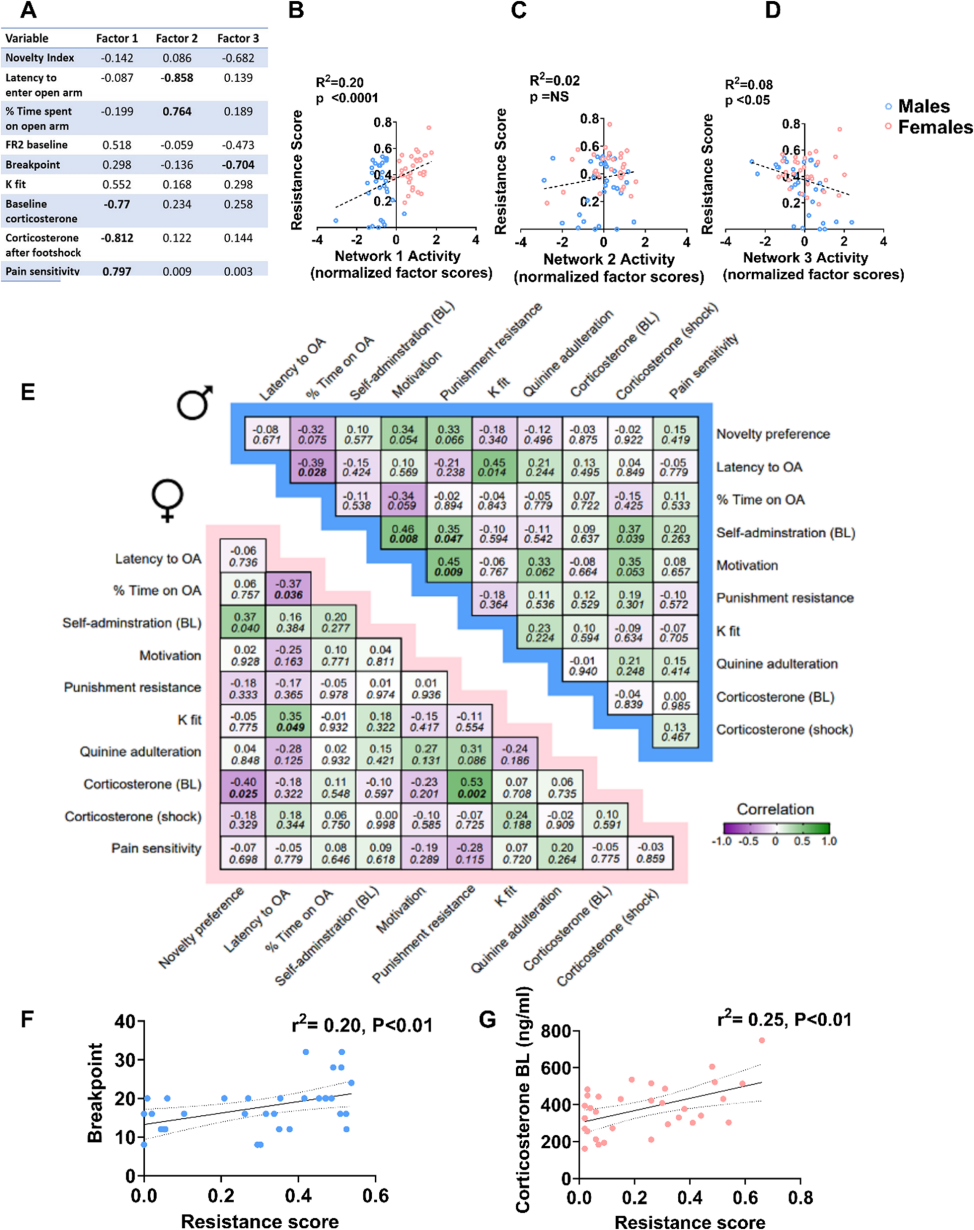
Compulsivity is correlated with motivation for alcohol in males and with corticosterone levels in females. **A** Factor loadings according to each factor. **B, C, D** Correlation analysis between activity of Factor 1, 2 and 3 generated through the factor analysis (see Results for definition) and resistance score. **E** Correlation table for individual primary variables for males (blue) and females (pink). **F**, **G** Correlational analysis between the resistance score, motivation and corticosterone levels in male and female rats, respectively. Values in two entry table are Pearson’s correlation values and *p* values (italic, below). Bold values are *p* values lower than 0.05. OA, open arms; BL, baseline

In search of predictors of compulsivity, male (*n* = 32) and female (*n* = 32) rats were additionally screened for novelty-induced place preference, anxiety-like behavior and corticosterone levels. A principal component extraction followed by factor analysis and multiple regression were carried out to model the relationships between these behaviors, corticosterone levels, and the battery of alcohol-related behaviors. Three factors with eigenvalues > 1 were obtained. Following normalized Varimax rotation, these collectively accounted for 61% of total variance. Corticosterone levels at both basal and after punishment conditions as well as pain sensitivity loaded on a “Factor 1” which accounted for 33% of total variance. Latency time and % time spent in open arms of the EPM loaded on a “Factor 2”; while motivation to self-administer alcohol loaded on a “Factor 3” that accounted for 15% and 13% of variance, respectively. Factor loadings are shown in Fig. 6A.

We then analyzed whether factor scores were associated with compulsivity, and whether this association differed by sex. Factor scores on Factor 1 and 3 but not Factor 2 showed a significant correlation with compulsivity (Fig. 6B–D, r^2^ = 0.2, *p* < 0.001, *r*^2^ = 0.02, *p* < 0.2, *r*^2^ = 0.08, *p* < 0.05). Multiple regression analysis using resistance score (0.2 mA) as the dependent variable in male and female rats showed that in males, compulsivity correlated significantly (*p* < 0.01) with the reward-motivation associated Factor 3, while in females, compulsivity correlated significantly with the stress-pain associated Factor 1 (*p* < 0.05).

Similar results were found when examining the relationship between compulsivity and the primary variables (Fig. 6E). In males but not in females, motivation to self-administer alcohol (number of progressive ratio breakpoints) showed a significant correlation with compulsivity, indexed as a resistance score (Fig. 6F; linear regression *r*^2^ = 0.17, *p* < 0.05) while no such correlation was found in females (*r*^2^ = 0.019, *p* = 0.45). Conversely, in females, there was a significant correlation between basal corticosterone levels and resistance to a 0.2-mA shock punishment (Fig. 6G; linear regression, *r*^2^ = 0.21, *p* < 0.01) while no such correlation was found in males (*r*^2^ = 0.013, *p* = 0.53). There were no significant differences according to sex in basal anxiety or novel place preference (Additional file 1: Fig. S3B, C).

### Estrous cycle has no effect on the alcohol-related behaviors studied

The influence of the estrous cycle (Additional file 1: Fig. S4) was assessed throughout the main experimental procedures. There were no significant differences between diestrus or proestrus phases (data not shown), thus we combined the diestrus and proestrus data as (non-estrous) for the statistical analysis as previously reported [40]. We acknowledge the fact that fluctuation of estradiol and progesterone levels across the cycle phases can represent a source of variability on the behavioral outcome. Thus, future studies that account for hormonal changes throughout the different estrous stages might provide a better understanding on the influence of each estrous phase on the alcohol-related behaviors.

Overall, we did not find a significant contribution of the different estrous phases on alcohol-related behaviors. The only significant effect was found on the resistance to quinine, with females in the non-estrus phase achieving a higher number of quinine-adulterated alcohol reinforcers (Table 1, one-way ANOVA, main effect of phase, *F*_(1,94)_ = 4.99; *p* < 0.05; *η*^2^ = 0.05).

**Table 1:** Estrus and non-estrus phase related to alcohol-related behavioral assessment

Behavior	Estrus Mean ± SEM	Non-estrus Mean ± SEM	Subject (*N*) estrus	Subject (*N*) non-estrus	Phase effect *F*-value, *p* value; *η*^2^
Alcohol self-administration (reinforcers)	14.81 (± 7.5)	15.05 (± 5.7)	53	107	*F*_1,158_ = 0.81, *p* = 0.57
Motivation (break points)	11.2 (± 4.3)	13.9 (± 6.12)	12	20	*F*_1,30_ = 1.4, *p* = 0.186
Footshock-punished alcohol self-administration (reinforcers)	6.3 (± 7.4)	5.6 (± 6.3)	82	76	*F*_1,156_ = 0.59, *p* = 0.6
Resistance to quinine-induced alcohol adulteration (reinforcers)	9.3 (± 3.7)	11.4 (± 5.1)	53	43	*F*_1,94_ = 2.34, *p < 0.05; *η*^2^ < 0.05
Temporal discounting of alcohol reward (reinforcers)	5.7 (± 1.8)	6.5 (± 2.9)	38	26	*T*_1,162_ = 1.34, *p* = 0.2
Anxiety (% time in open arms)	65.5 (± 16.6)	68.1 (± 14.8)	14	18	*T*_1,130_ = 0.47, *p* = 0.6
Pain sensitivity (mA) (shock threshold)	0.2 (± 0.03)	0.2 (± 0.05)	14	17	*T*_1,29_ = 0.47, *p* = 0.6

## Discussion

We carried out an extensive battery of tests to examine potential sex differences in alcohol-related behaviors in Wistar rats. Overall, we did not find sex differences in body weight-corrected self-administration under fixed ratio, unpunished self-administration conditions. In contrast, males exhibited a stronger motivation to obtain alcohol, while females displayed a higher compulsive alcohol self-administration, associated with higher corticosterone levels at baseline condition.

Our findings align with existing evidence, indicating that males generally exhibit higher rates of alcohol-reinforced responses in fixed ratio self-administration paradigms, but that intake levels are comparable when alcohol intake is expressed in (g/kg) [19]. In line with this observation, female rats drink more alcohol in minimal workload conditions (i.e., voluntary drinking in the home cage), whereas no sex differences are observed when some work (i.e., lever pressing) is required to obtain access to alcohol [17, 44]. The literature on operant alcohol self-administration has yielded mixed results, with some studies reporting no difference between males and females [17], while others have shown higher self-administration rates in females [19, 45, 46].

Our Wistar males showed a greater motivation to respond for alcohol compared to females, similar to what was recently shown by others [47]. In contrast, under similar progressive ratio conditions and alcohol concentration, no sex differences in break points were found in Long-Evans [19] or alcohol-preferring P-rats [16], suggesting the possibility of a strain-dependent effect. However, the lack of sex differences in the negative studies cited may result from a floor effect, due to the low breakpoint number achieved, in the Long-Evans rats [19], or a ceiling effect in the genetically selected alcohol-preferring rats, that show a higher motivation for alcohol compared to Wistars [48, 49].

Female rats showed an overall higher level of compulsive alcohol self-administration, a behavior operationally defined as self-administration that continues despite footshock punishment [29, 30, 50, 51] or quinine-induced alcohol adulteration [42, 52, 53]. This is consistent with recent reports of a greater resistance to footshock and quinine punished alcohol self-administration that has been recently shown in C57BL/6J female mice compared to males [24, 54–56]. These findings also generalize to another model of compulsive-like drinking, induced by alcohol deprivation [57]. A higher punishment resistance in females seems to be specific to alcohol, as both males and females have been shown to suppress their behavior for punished sucrose responding equally [54]. Moreover, females showed higher sensitivity to shock and to quinine-induced water adulteration showing that increased punishment resistance is not due to low sensitivity to pain or taste aversion per se.

Both footstock-punished self-administration and quinine adulteration reflect aversion-resistance, but rely on aversive stimuli that differ in sensory and possibly also affective properties. Despite this, we found that shock resistance and resistance to quinine adulteration loaded on the same factor, in line with our previous finding that shock-resistant alcohol self-administration and resistance to quinine adulteration are highly correlated [29]. This provides additional support for the existence of aversion-resistant alcohol intake as a shared underlying behavioral dimension.

Previous findings on individual variation in punishment-resistant alcohol self-administration were obtained in male rats only [29, 31]. Here, we expanded this characterization to females, and to a broader range of shock intensities. Alcohol-reinforced responding under conditions of contingent shock-punishment showed bimodal distributions in male but not in female rats when intermediate shock intensities were used (0.2 and 0.25 mA). Both male and females were unimodally distributed under low (0.1 mA) and high punishment conditions (0.3 and 0.35 mA), with males showing more stable measures of resistance to punishment over time.

The emergence of compulsivity, operationally defined as continued use despite negative consequences, has been proposed to reflect a shift from goal directed to habitual behavior [21]. A testable prediction that results from this conceptualization is that that animals showing compulsive alcohol self-administration should be less sensitive to outcome value than those that do not. We therefore systematically devalued alcohol by delaying its delivery, and generated a summary measure of the steepness with which the value of the alcohol rewards was discounted. This differed between males and females, but not between compulsive and non-compulsive animals within the respective sex. A less steep discounting observed in females could be interpreted as a persistent habitual response despite delay-induced reinforcer devaluation or as delayed gratification which requires a sustained interest and motivation in a delayed reward, and thereby more self-control. This might imply differences in cognitive control for alcohol rewards that are distant in time.

Our factor analysis suggests that an underlying behavioral dimension related to stress and pain predicted compulsivity in females.

While the impact of pain and stress on alcohol addiction is widely acknowledged, there is limited understanding of their intersection and the underlying neuroendocrine mechanisms. Clinical research has reported a negative correlation between high cortisol levels and pain thresholds induced by electric stimulation, hinting at a potential link between heightened stress and increased pain perception [58], as well as alcohol consumption [59]. Moreover, it has been found that higher cortisol levels induce visceral pain in women but not in men [60]. In the present study, we found an underlying behavioral dimension encompassing corticosterone levels and pain sensitivity to predict compulsive alcohol self-administration in females. One hypothesis might be that heightened corticosterone levels and pain sensitivity in females might promote compulsive drinking as a coping mechanism through negative reinforcement.

In contrast, compulsivity in males was predicted by reward-associated factors. These findings were further confirmed by simple correlation analyses of the individual underlying variables, where alcohol self-administration and motivation to self-administer alcohol correlated positively with punishment-resistance in males, but not in females.

Similarly, a recent investigation found a significant correlation between alcohol consumption and aversion-resistant alcohol intake in male Lister Hooded rats. The same study reported considerable individual differences in AUD-associated behavioral constructs underscoring the importance of accounting for individual heterogeneity in AUD-like behaviors [61].

In females, punishment resistance was predicted by higher corticosterone levels. Although motivation and resistance to punishment are often associated [62], the relationship between these measures is not straightforward. In fact, studies have found that punishment resistance and PR responding do not always correlate [63–66], suggesting that the willingness to exert an effort or the willingness to tolerate a negative outcome to obtain a reward might represent overlapping behavioral domains. Our data indicate that the nature of this overlap may be different between males and females, with positively reinforcing factors dominating in males, and factors reflecting negatively reinforcement having a greater influence in females.

These findings of sex-differences are in line with clinical observations. For instance, alcohol-dependent women are more likely to suffer stress-related psychiatric comorbidities such as anxiety and mood disorders than men [2, 67]. Women are also more likely to drink heavily to alleviate unpleasant emotions [4–6], and to relapse in response to negative affect [7]. In contrast, men are more likely to report drinking due to social factors, and in response to positive emotions [8–10]. They are also more likely to report positive moods prior to [8] and during relapse [68].

Finally, consistent with previous studies showing that the estrous cycle does not substantially impact alcohol intake in naturally cycling rats [16, 69], we did not find significant difference in alcohol-related behaviors across the stages of the estrous. This is consistent with previous alcohol models where females cohabitated in the same housing room as males and estrous cycle had no effect on alcohol drinking in any strain or drinking model [17].

### Perspective and significance

Preclinical models are essential for understanding the behavioral and neurobiological mechanisms of alcohol addiction. Incorporating sex as a biological factor is fundamental for gaining a better understanding of vulnerability factors in the development of alcohol addiction, and has the potential to guide treatment choices to make them better tailored to the needs of the individual patient. Our dataset provides a comprehensive overview of how sex-differences influence alcohol-related behaviors, offering valuable insights to advance both preventive measures and treatment strategies within clinical settings.

## Conclusions

In conclusion, we present a broad analysis of alcohol-related behaviors with a focus on compulsivity, its sex-dependence, and its antecedents. Our findings suggest that mechanisms underlying compulsive alcohol self-administration differ between males and females. This observation highlights the need to include sex as biological variable in both preclinical and clinical research, and has potential treatment implications in alcohol addiction.

### Supplementary Information

**Additional file 1: Figure S1**. Alcohol self-administration under 0.1 and 0.35 mA punishment is unimodally distributed in male and female rats. Resistance score distribution of punished alcohol self-administration across the 3 last days color coded for males (blue) and females (red) under** A**) 0.1 mA and **B**) 0.35 mA shock intensity. **Figure S2**. (**A**) Resistance score across 10 days for males (blue, top) and females (pink, bottom) under 0.1–0.35 mA shock intensities. Each line is an individual animal. (**B)** Lower persistence score value (StDev) to resistance to punishment in males compared to females under 0.2 and 0.25 mA shock intensities, indicating higher consistency in their response (p < 0.001**). **Figure S3**. (**A**) Shock sensitivity differed between male and female rats. Mean (± SEM) footshock threshold between male (n = 16) and female (n = 16) rats. F_1, 30_ = 23.9, p < 0.001; η^2^ = 0.44 p < 0.001*** males vs females. (**B, C**) Basal anxiety-like behavior and novelty preference did not differ between male (n = 32) and female (n = 32) rats. Mean (± SEM) percentage time spent in the open arm (F_1, 62_ = 3.71, p = 0.06) and in the novel compartment (F_1, 62_ = 2.59, p = 0.1). **Figure S4**. Photomicrographs of vaginal smear of rats showing four phases of estrous cycle. (**P**) Proestrous phase: nucleated epithelial cells, (**E**) Estrous phase: non- nucleated cornified cells, (**D**) Diestrous phase: leukocytes. (**M**) Metestrous phase: nucleated epithelial cells, non- nucleated cornified cells and leukocytes.

## Data Availability

The datasets used and/or analyzed during the current study can be made available from the corresponding author on reasonable request.
