# Supplementary material for: Different mechanisms underlie compulsive alcohol self-administration in male and female rats
Source: Biol Sex Differ. 2024 Feb 17;15:17. doi: 10.1186/s13293-024-00592-5 (PMC10874042; doi:10.1186/s13293-024-00592-5)
Supplement: Supplementary file 1 — Additional file 1: Figure S1. Alcohol self-administration under 0.1 and 0.35 mA punishment is unimodally distributed in male and female rats. Resistance score distribution of punished alcohol self-administration across the 3 last days color coded for males (blue) and females (red) under A) 0.1 mA and B) 0.35 mA shock intensity. Figure S2. (A) Resistance score across 10 days for males (blue, top) and females (pink, bottom) under 0.1–0.35 mA shock intensities. Each line is an individual animal. (B) Lower persistence score value (StDev) to resistance to punishment in males compared to females under 0.2 and 0.25 mA shock intensities, indicating higher consistency in their response (p < 0.001**). Figure S3. (A) Shock sensitivity differed between male and female rats. Mean (± SEM) footshock threshold between male (n = 16) and female (n = 16) rats. F1, 30 = 23.9, p < 0.001; η2 = 0.44 p < 0.001*** males vs females. (B, C) Basal anxiety-like behavior and novelty preference did not differ between male (n = 32) and female (n = 32) rats. Mean (± SEM) percentage time spent in the open arm (F1, 62 = 3.71, p = 0.06) and in the novel compartment (F1, 62 = 2.59, p = 0.1). Figure S4. Photomicrographs of vaginal smear of rats showing four phases of estrous cycle. (P) Proestrous phase: nucleated epithelial cells, (E) Estrous phase: non- nucleated cornified cells, (D) Diestrous phase: leukocytes. (M) Metestrous phase: nucleated epithelial cells, non- nucleated cornified cells and leukocytes. [file 13293_2024_592_MOESM1_ESM.docx]

**Supplementary Information**


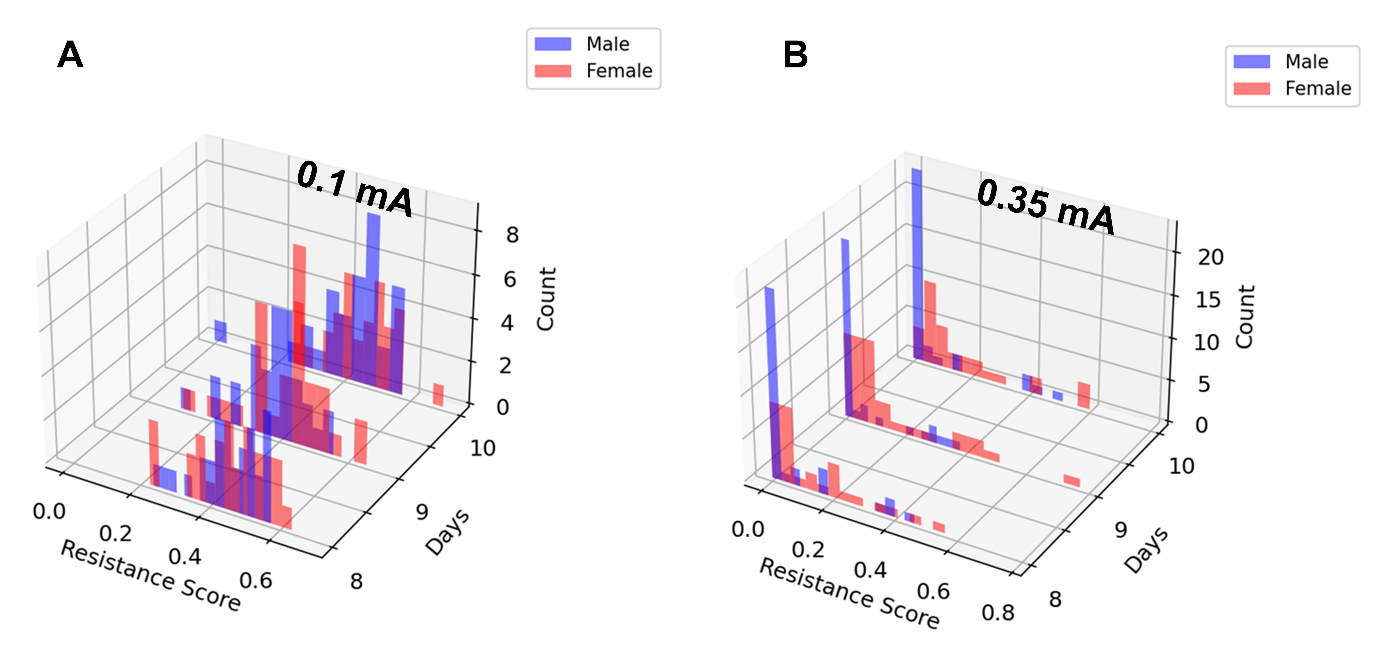


**Figure S1**. **Alcohol self-administration under 0.1 and 0.35 mA punishment is unimodally distributed in male and female rats**. Resistance score distribution of punished alcohol self-administration across the 3 last days color coded for males (blue) and females (red) under **A**) 0.1 mA and **B**) 0.35 mA shock intensity.


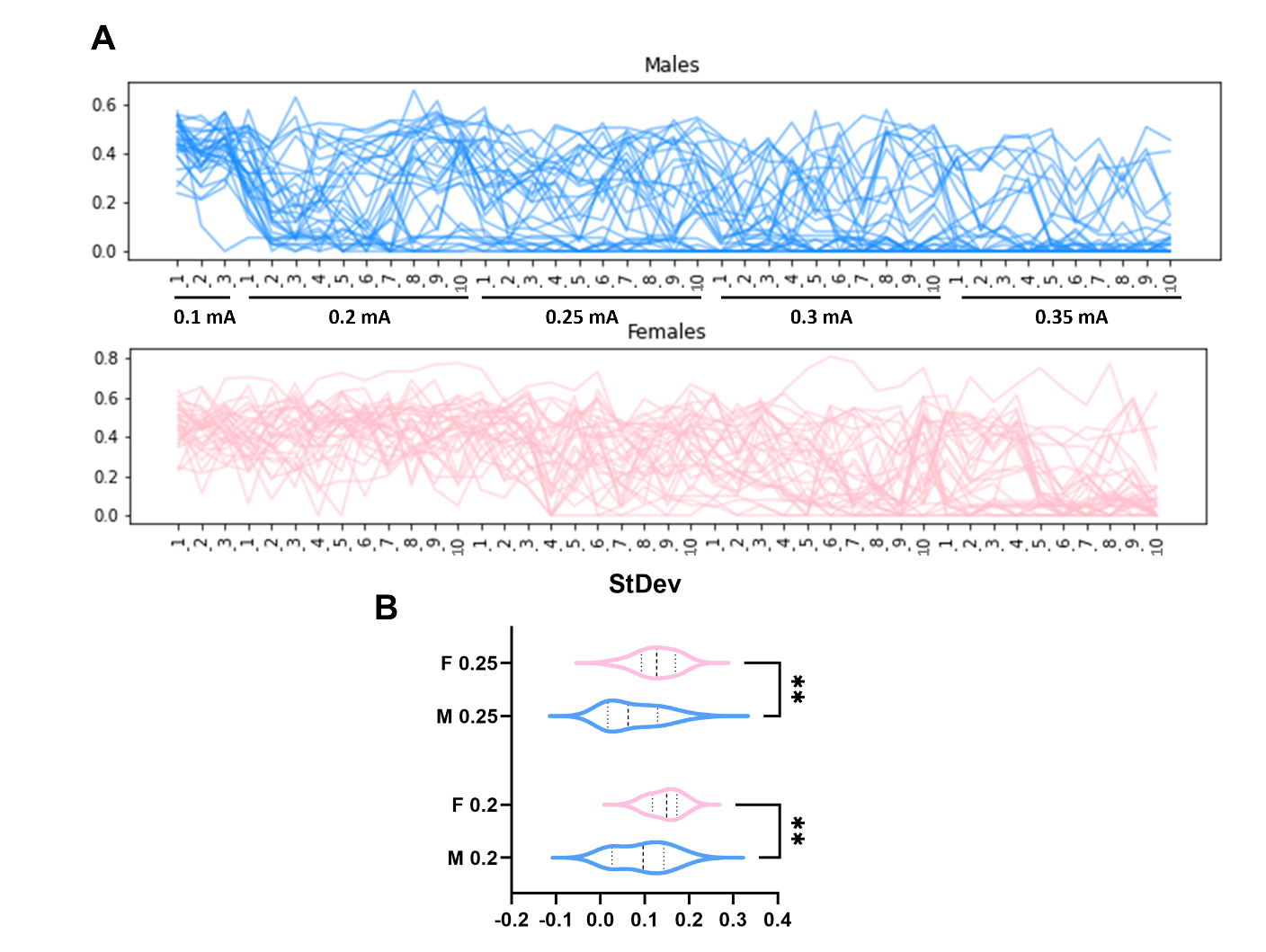


**Figure S2**. (**A**) Resistance score across 10 days for males (blue, top) and females (pink, bottom) under 0.1-0.35 mA shock intensities. Each line is an individual animal. (**B)** Lower persistence score value (StDev) to resistance to punishment in males compared to females under 0.2 and 0.25 mA shock intensities, indicating higher consistency in their response (p<0.001**).

**Figure S3**. (**A**) Shock sensitivity differed between male and female rats. Mean (±SEM) footshock threshold between male (n=16) and female (n=16) rats. F_1, 30_= 23.9, p<0.001; η^2^= 0.44 p<0.001*** males vs females. (**B, C**) Basal anxiety-like behavior and novelty preference did not differ between male (n=32) and female (n=32) rats. Mean (±SEM) percentage time spent in the open arm (F_1, 62_= 3.71, p=0.06) and in the novel compartment (F_1, 62_= 2.59, p=0.1).

**
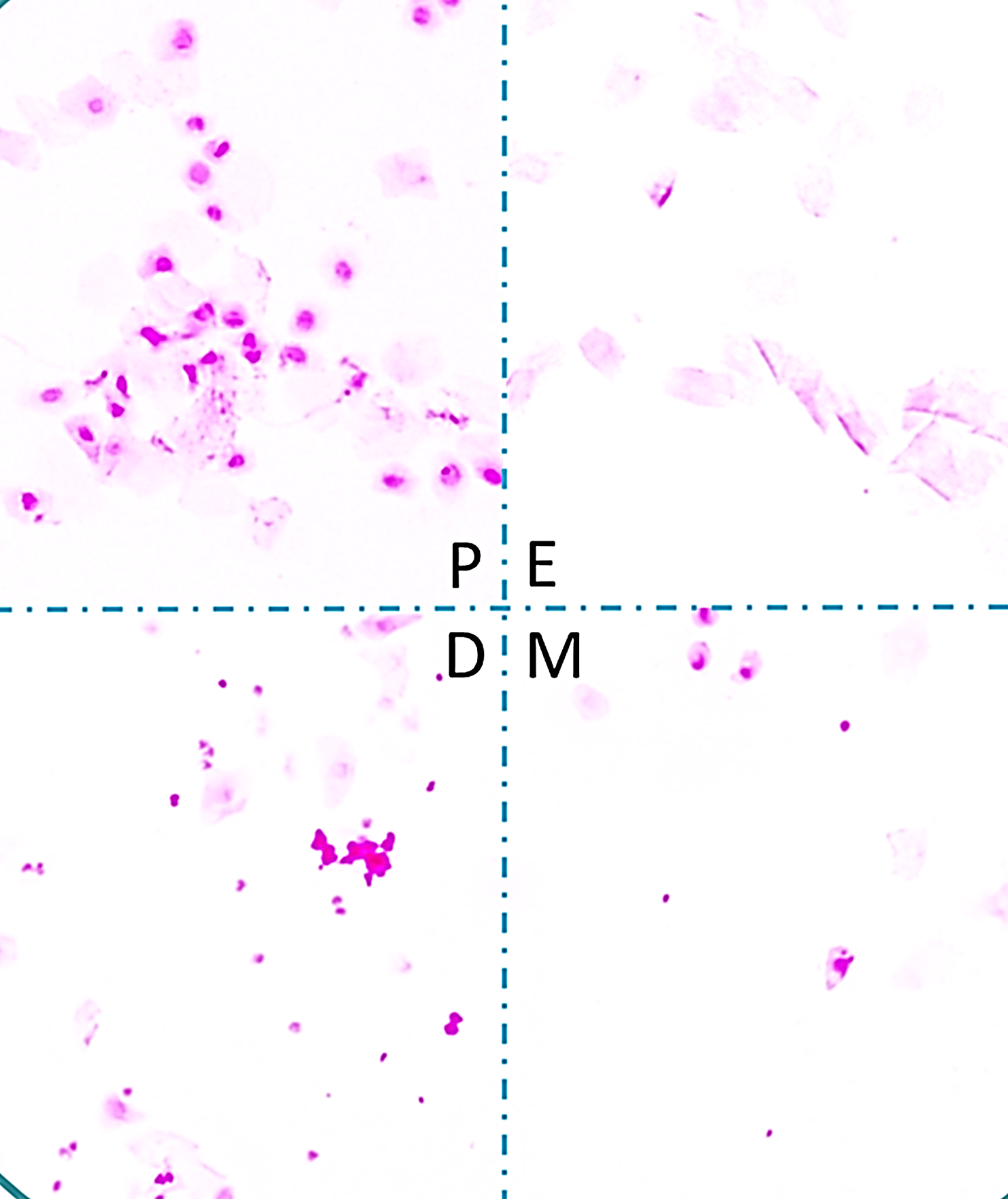
**

**Figure S4**. Photomicrographs of vaginal smear of rats showing four phases of estrous cycle. (**P**) Proestrous phase: nucleated epithelial cells, (**E**) Estrous phase: non- nucleated cornified cells, (**D**) Diestrous phase: leukocytes. (**M**) Metestrous phase: nucleated epithelial cells, non- nucleated cornified cells and leukocytes.
